# Beyond phoresy: ecological patterns, effects of host attributes, and temporal variation in mites associated with *Rhynchophorus palmarum* L. (Coleoptera: Curculionidae)

**DOI:** 10.1007/s10493-026-01171-6

**Published:** 2026-08-24

**Authors:** Maria Beatriz Nunes-Souza, Maria Eduarda Barbosa Almeida, Erika Luiza dos Santos Torres, Hans Klompen, Manoel Guedes Correa Gondim Junior, Debora Barbosa de Lima, José Wagner Silva Melo

**Affiliations:** 1 Laboratório de Acarologia, Departamento de Zoologia, Programa de Pós-Graduação em Biologia Animal, Universidade Federal de Pernambuco, Recife, Pernambuco Brazil; 2 Laboratório de Acarologia, Departamento de Zoologia, Graduação em Ciências Biológicas com ênfase em Ciências Ambientais, Universidade Federal de Pernambuco, Recife, Pernambuco Brazil; 3 Department of Evolution, Ecology, and Organismal Biology, The Ohio State University, Columbus, OH 43212 USA; 4 Laboratório de Acarologia, Departamento de Agronomia, Programa de Pós-Graduação em Entomologia, Universidade Federal Rural de Pernambuco, Recife, Pernambuco Brazil

**Keywords:** Community, Aggregation pattern, Microhabitat, Competitive exclusion, Niche partitioning, South American Palm Weevil

## Abstract

Phoresy, a common dispersal strategy among arthropods, involves complex relationships between hosts and symbionts that influence ecological dynamics. Investigating the structure of phoretic mite communities and their population fluctuations is particularly relevant for widely distributed agricultural pests, such as the coconut palm weevil, *Rhynchophorus palmarum*. This study investigated the community of phoretic mites associated with *R. palmarum* in its native Neotropical range, aiming to characterize population patterns, evaluate the influence of host attributes, and analyze interspecific dynamics. A total of 14 mite taxa were recorded, all exhibiting high prevalence and strong aggregation. The mite *Centrouropoda* cf. *brasiliana* stood out as the dominant species, with individual loads exceeding 2,800 mites per beetle. Although host sex and body size did not affect total phoretic load, body size selectively influenced the abundance of certain taxa (*Lasioseius* sp., *Macrocheles mammifer*, Gamasina sp. 1, and *Crenamargo binuseta*). The temporal variation of *C.* cf. *brasiliana* were negatively modulated by *C. binuseta* and *Rhynchopolipus rhynchophori*, suggesting mechanisms of competitive exclusion or microhabitat partitioning. These findings highlight a complex community in which biotic interactions play a central role in structuring the phoretic acarifauna of *R. palmarum*, emphasizing the need for further investigations into the functional effects of these relationships.

**Supplementary Information:**

The online version contains supplementary material available at https://doi.org/10.1007/s10493-026-01171-6.

## Introduction

Phoresy is a dispersal strategy widely employed by small, wingless organisms, such as mites, which use larger and more mobile organisms to colonize new habitats, generally without establishing a parasitic relationship (Athias-Binche and Morand 1993; Krantz and Walter 2009; Seeman and Walter 2023). This interaction is common among mites belonging to Astigmata, Heterostigmata, Mesostigmata (Schwarz et al. 1998; Seeman and Walter 2023). Although morphological adaptations to phoresy are well documented, the ecological factors regulating species composition, prevalence, and coexistence in phoretic communities remain poorly explored, especially in tropical ecosystems, where the diversity of these mites is still underestimated.

The beetle *Rhynchophorus palmarum* L., 1764 (Coleoptera: Curculionidae) has a wide distribution in the Neotropical region and is recognized for its agricultural importance as the main vector of the nematode *Bursaphelenchus cocophilus* (Cobb, 1919) Baujard, the causal agent of red ring disease in coconut (*Cocos nucifera* L.) and oil palm (*Elaeis guineensis* Jacq.) (Griffith 1968; Giblin-Davis et al. 2003). Despite its economic relevance and extensive distribution in Central and South America, studies on the phoretic acarifauna associated with *R. palmarum* remain scarce and fragmented. Most available information is limited to isolated taxonomic records or incidental observations (Husband and Flechtmann 1972; Flechtmann 1981; Di Palma and Porcelli 2014; Gómez-Marco et al. 2021), even within its area of origin (Rodríguez-Morell et al. 2012).

Considering the high diversity of mites associated with insects and the aggregation patterns frequently observed, it is plausible that phoretic communities on *R. palmarum* are structured not only by host attributes, such as sex and body size (Dilipkumar et al. 2015; Gómez-Marco et al. 2021), but also by interspecific interactions, such as competitive exclusion and spatial partitioning of microhabitats. The differential use of host body regions may facilitate coexistence among species, whereas asymmetries in prevalence and abundance may reflect dominance hierarchies or antagonistic associations (Matos et al. 2023). However, studies investigating these dynamics are rare, especially those focusing on temporal variation in abundances, which limits our understanding of the factors regulating stability and coexistence in phoretic communities. Evaluating temporal patterns is essential to detect biotic interactions subtly modulated by environmental variation or by fluctuations in the density of coexisting species. Furthermore, such investigations allow the identification of consistent patterns of dominance, competitive exclusion, or resource partitioning. In a context where phoretic taxa may include commensal, mutualistic, or even facultative parasitic forms (Houck and Cohen 1995; Seeman and Walter 2023), understanding their dynamics over time is crucial to elucidate the functional role of these associations.

In the present study, a survey of phoretic mites associated with *R. palmarum* was conducted in a commercial coconut plantation in Northeastern Brazil, a region of notable agricultural importance located within the natural distribution range of the beetle. This study aimed to: (i) characterize the phoretic mite community by evaluating patterns of prevalence, intensity, abundance, and aggregation; (ii) investigate the effects of host sex and body size on total and species-specific phoretic load; and (iii) examine potential interspecific interactions affecting temporal changes in the abundance of the most dominant phoretic mite species.

## Methodology

### Study area and collection of Rhynchophorus palmarum specimens

The study was conducted in a coconut plantation located in Igarassu, Pernambuco (7°50’38.8"S 34°56’28.3"W), from December 2023 to December 2024. Sampling was carried out every two weeks (17 days), totaling 18 sampling events. *R. palmarum* beetles were captured using traps, as described by Ferreira et al. (2002). Each trap consisted of a polyethylene bucket (1.5 L) with a perforated lid to allow insect entry, containing a microtube with synthetic aggregation pheromone (rhynchophorol, 6-methyl-2-(E)-hepten-4-ol, Interacta Química) and semi-macerated sugarcane stalks as a food attractant. Traps were randomly distributed across the plantation area, fixed next to the coconut palm stipe at ground level, and spaced at least 100 m apart.

### Insect morphometry and extraction of mite specimens

Captured beetles were individually placed in Falcon tubes (50 mL), without alcohol, and transported to the Acarology Laboratory of the Federal University of Pernambuco (UFPE), where they were stored at − 30 °C until processing. In the laboratory, individuals were counted, sexed, and measured for body length (from the head to the pygidium (adapted from Huang et al. 2018), in dorsal view—an indicator of reproductive performance, such as fecundity and mating capacity in coleopterans, according to Huang et al. 2018) and width (at the widest point, in dorsal view—an indicator of longevity in coleopterans, according to Damron et al. 2021). Measurements were taken using a digital caliper (Digital Caliper Fractional and Decimal Display, Neiko Tools USA). Damaged beetles (with missing body parts) were discarded. Attached mites were removed using a brush, forceps, and scalpel, counted, separated into morphospecies, and preserved in 70% ethanol for later identification.

### Mounting and identification of mite specimens

Sclerotized specimens were cleared in Nesbitt’s solution (Krantz and Walter 2009) for seven days, whereas weakly sclerotized specimens were mounted directly on slides using Hoyer’s medium, between slide and coverslip, following Krantz and Walter (2009). Slides were kept in an oven at 50 °C for seven days and, after drying, were sealed with varnish (Acrilex^®^). Mites were examined under a phase-contrast microscope (Olympus^®^ BX41) for identification to the lowest possible taxonomic level, with the aid of existing literature (Krantz and Walter 2009; Di Palma and Porcelli 2014), as well as contributions from specialists in specific mite groups. Voucher specimens were deposited in the Mite Collection of the Acarology Laboratory at the Universidade Federal de Pernambuco (UFPE), Recife, Brazil (8°03’00.8"S, 34°56’52.2"W), under the following accession numbers: Blattisociidae, Lot 1 LA-UFPE 00002–00011; Macrochelidae, Lot 1 LA-UFPE 00001–00020; Diplogyniidae, Lot 1 LA-UFPE 00001–00010; Urodinychidae, Lot 1 LA-UFPE 00001–00010; Uropodidae, Lot 1 LA-UFPE 00001–00010; Trematuridae, Lot 1 LA-UFPE 00001–00010; Podapolipidae, Lot 1 LA-UFPE 00001–00010; Acaridae, Lot 1 LA-UFPE 00001–00010; Histiostomatidae, Lot 1 LA-UFPE 00001–00010; Dinychidae, Lot 1 LA-UFPE 00001–00010; Gamasina, Lot 1 LA-UFPE 00001–00010; and Uropodina (including *Centrouropoda* cf. *brasiliana*), Lot 1 LA-UFPE 00001–00020.

### Temporal variation in the abundance of dominant phoretic mites

Temporal variation in the abundance of dominant phoretic mite species were investigated only for mite species considered dominant, classified based on the species importance index (SII), calculated as: SII = prevalence (%) × mean abundance (%). Species were categorized as dominant (SII ≥ 100), common (10 < SII < 100), or rare (SII < 10), following Song et al. (2019). Mean mite abundance per beetle (biotic variable) and minimum, mean, and maximum temperature data (°C), as well as mean precipitation (mm) (abiotic variables), were used as predictors of the temporal variation in the abundance of dominant phoretic mite species. Meteorological data were obtained from the Agrometeorological Monitoring System (AGRITEMPO), TRMM.6729 station (Igarassu, Pernambuco), considering the average values of the five days during which the traps remained in the field.

### Data analysis

Initially, descriptive metrics were calculated for each mite taxon: prevalence (proportion of infested beetles), mean intensity (average number of mites considering only infested hosts), mean abundance (average number of mites per beetle, considering all individuals), degree of aggregation (measured by Poulin’s D index), and range (minimum and maximum number of mites per host). All these metrics, except for range, were compared using 95% confidence intervals. For prevalence, confidence intervals were estimated using the exact Clopper–Pearson method, whereas for the remaining metrics they were obtained by bootstrap (2,000 replicates; bias-corrected and accelerated adjustment). To assess the influence of beetle sex on total and taxon-specific phoretic load, the same approaches described above were applied, but restricted to prevalence, intensity, and abundance metrics, calculated separately for male and female beetles. Each metric provides a distinct perspective on the mite–beetle interaction: prevalence indicates whether both sexes are equally used as hosts, reflecting usage patterns; intensity reveals whether, among infested individuals, one sex carries higher phoretic loads; and abundance allows the evaluation of differences in the total number of mites transported between sexes, considering both infested and non-infested hosts.

The influence of body size on phoretic load was analyzed using generalized linear models (GLMs) with a quasi-Poisson distribution, appropriate for count data with overdispersion. As a measure of body size, estimated body area (length × width) was used, assuming correlation with mite attachment surface. Species rarity classifications were presented using species rank plots. The most dominant mite species had its temporal variation analyzed using a stepwise multiple linear regression, with biotic and abiotic variables as predictors. Model assumptions (homogeneity of variance, linearity, normality of residuals, absence of influential observations, and low collinearity) were evaluated graphically and using the variance inflation factor (VIF) (excluding variables with VIF > 5). Final model selection considered R², F, and P values, as well as the relative contribution of variables (retaining only those explaining ≥ 5% of R²). The relative importance of predictors was estimated using the “lmg” method. All analyses were performed in the R language.

## Results

**Fig. 1:**
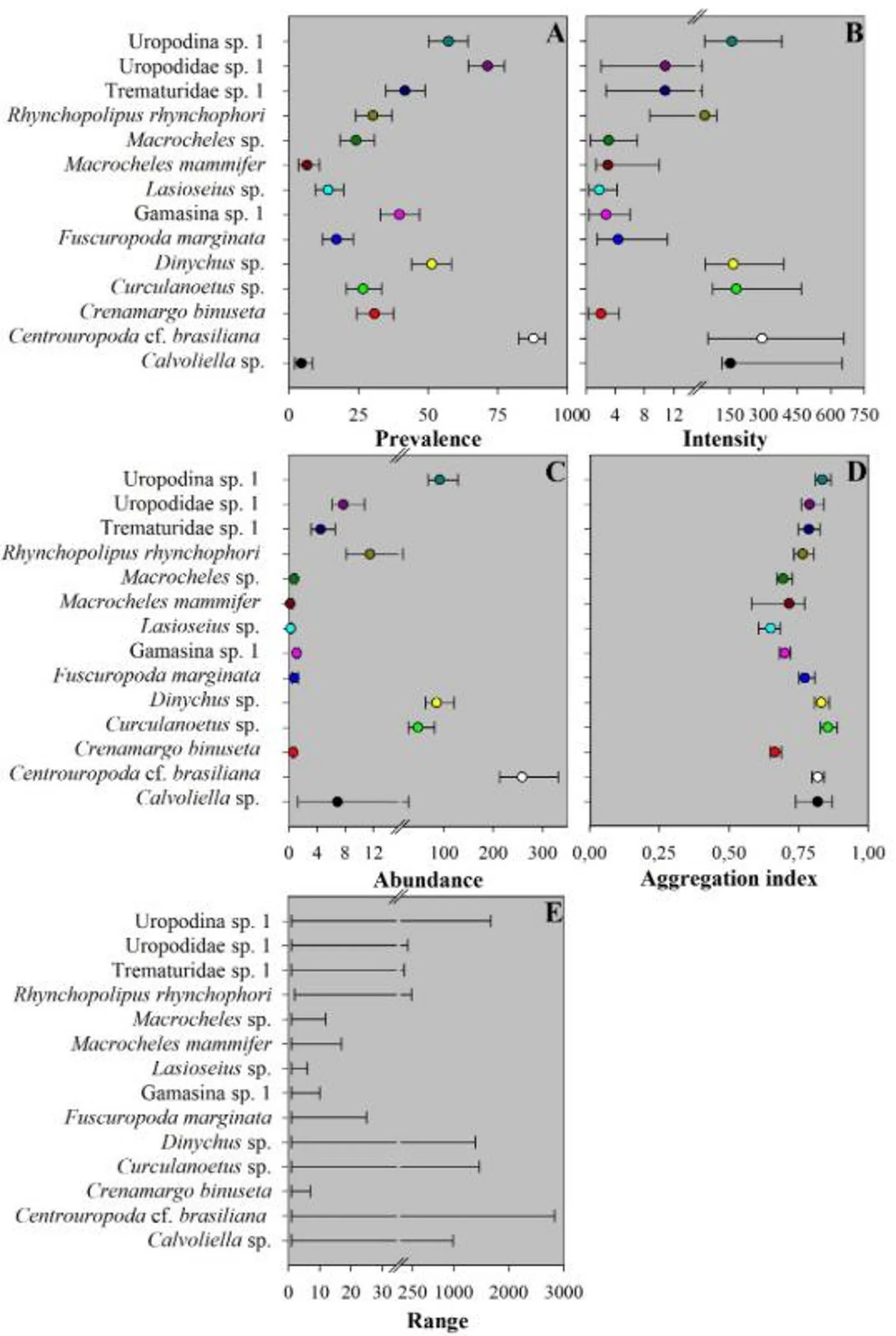
Descriptive infestation metrics of mites associated with *Rhynchophorus palmarum*, including prevalence (**A**), mean intensity (**B**), mean abundance (**C**), degree of aggregation (**D**), and range (**E**) for each recorded taxon. Points represent mean values, whereas bars correspond to 95% confidence intervals, except for infestation range, where bars represent minimum and maximum values (mites/beetle)

Fourteen mite taxa were recorded associated with 198 of the 199 beetles collected, namely: *Fuscuropoda marginata* (Koch, 1839) (Urodinychidae), *Macrocheles mammifer* Berlese, 1918 (Macrochelidae), *Crenamargo binuseta* Hicks, 1958 (Diplogyniidae), *Rhynchopolipus rhynchophori* (Ewing, 1924) (Podapolipidae), *Centrouropoda* cf. *brasiliana* Wisniewski & Hirschmann, 1992 (Uropodina s.l.), *Dinychus* sp. Kramer, 1886 (Dinychidae), *Macrocheles* sp. Latreille, 1829 (Macrochelidae), *Lasioseius* sp. Berlese, 1916 (Blattisociidae), *Curculanoetus* sp. Fain, 1974 (Histiostomatidae), *Calvoliella* sp. Samsinak, 1969 (Acaridae), Gamasina sp. 1, Trematuridae sp. 1, Uropodidae sp. 1, and Uropodina sp. 1. These taxa exhibited wide variation in prevalence, intensity, abundance, and degree of aggregation (Fig. 1).

Species prevalence ranged from 5% to 88%. The mite *C.* cf. *brasiliana* showed the highest prevalence (88%), followed by Uropodidae sp. 1 (71%) and Uropodina sp. 1 (57%). Seven taxa exhibited intermediate prevalence (24–51%), whereas *Calvoliella* sp., *M. mammifer*, *Lasioseius* sp., and *F. marginata* were the taxa with the lowest prevalence (below 20%) (Fig. 1A). Mean infestation intensity varied widely among taxa. The mites *C.* cf. *brasiliana*, *Curculanoetus* sp., *Dinychus* sp., Uropodina sp. 1, and *Calvoliella* sp. showed high values (≥ 150 mites per infested beetle), indicating intense infestations when present. In contrast, *R. rhynchophori*, Uropodidae sp. 1, and Trematuridae sp. 1 exhibited moderate intensities (10–40), whereas the remaining taxa showed low intensity (below 10) (Fig. 1B). Regarding mean abundance, *C.* cf. *brasiliana* stood out with an average of 258.64 individuals per beetle. The mites *Dinychus* sp. and Uropodina sp. 1 also showed high values (> 85), whereas most taxa with low intensity exhibited abundance values below 1 (Fig. 1C). Regarding the degree of aggregation, all taxa showed high values of Poulin’s discrepancy index (D ≥ 0.65), particularly *Curculanoetus* sp. (0.86), *Dinychus* sp. (0.83), and Uropodina sp. 1 (0.84). These values indicate strong aggregation: most beetles carry few or no mites, whereas a minority harbor large phoretic loads (Fig. 1D). The range further reinforces this pattern, varying from few individuals (none or one) to thousands per host. The mite *C.* cf. *brasiliana* showed the greatest variation, with up to 2,834 individuals on a single beetle, whereas *Calvoliella* sp., the taxon with the lowest prevalence, reached maximum loads of up to 980 mites per individual (Fig. 1E).

### Effects of host attributes on phoretic load

**Fig. 2:**
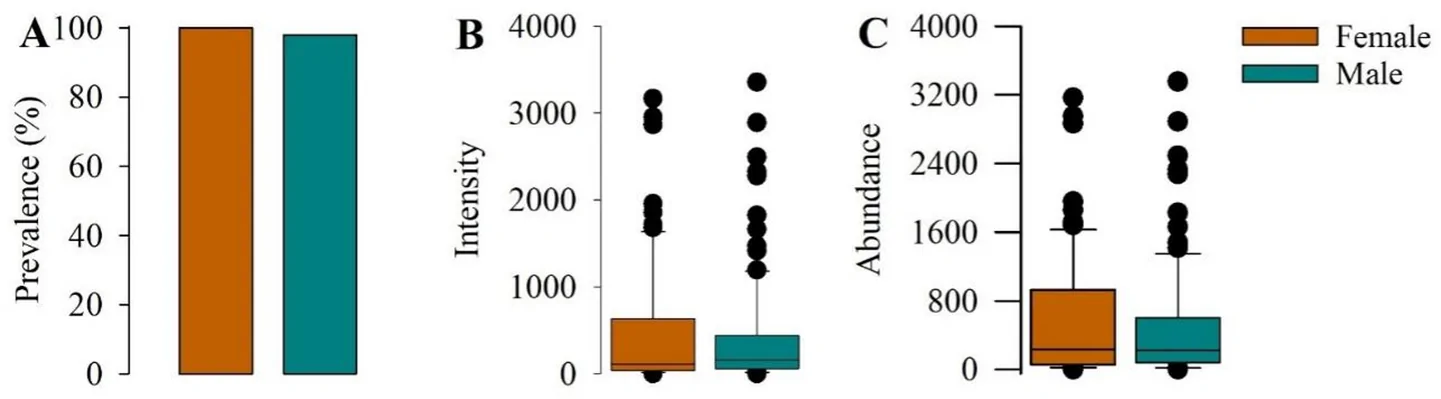
Boxplots comparing prevalence (**A**), intensity (**B**), and abundance (**C**) of the total phoretic load of mites associated with female and male *Rhynchophorus palmarum*. Each point represents an individual; boxes indicate the interquartile range (Q1–Q3), internal lines correspond to the median, and extremes represent outliers. No significant differences were observed between sexes for any of the metrics

No differences were detected between male and female *R. palmarum* in relation to phoretic load, either overall (considering all taxa combined) or specifically for each taxon. The three evaluated metrics (prevalence, intensity, and abundance) showed similar values between sexes, with overlapping confidence intervals. Prevalence was 100% in females and 99.1% in males, mean intensity was 575.2 and 476.5 mites per infested beetle, respectively, and mean abundance was 575.2 and 472.2 mites per beetle, respectively. These results indicate that males and females are used equivalently as phoretic hosts by the different mite groups (Fig. 2). Detailed data for each taxon are provided in Supplementary Table 1.

**Fig. 3:**
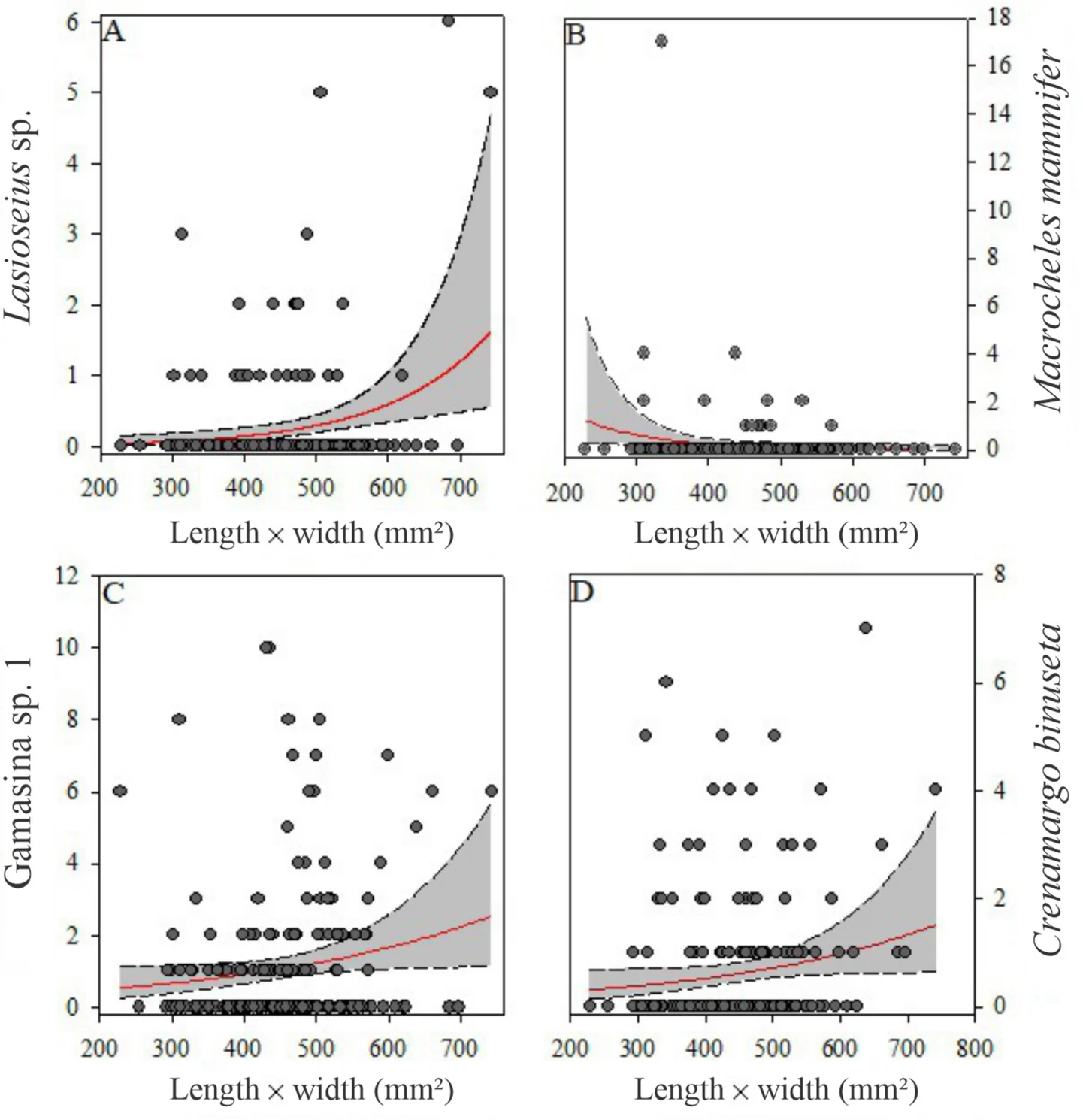
Relationship between a proxy of body area of *Rhynchophorus palmarum* (length × width) and the abundance of different taxa of phoretic mites. Red lines represent central trends, whereas shaded areas delimited by dashed black lines indicate 95% confidence intervals. (**A**) *Lasioseius* sp.; (**B**) *Macrocheles mammifer*; (**C**) Gamasina sp. 1; (**D**) *Crenamargo binuseta*. *n* = 198 beetles

The body length of the collected *R. palmarum* specimens ranged from 27.40 to 45.60 mm (35.6 ± 0.33 mm, mean ± SE), whereas pronotum width ranged from 8.80 to 16.70 mm (12.7 ± 0.11 mm, mean ± SE). Insect body dimensions (length × width) showed no significant relationship with total mite load (β = 3.2 × 10⁻⁶; *p* = 0.998), indicating that larger beetles do not, on average, carry more mites than smaller individuals (Table 1). When considered separately, most taxa also showed no significant association with body size. Only *Lasioseius* sp., Gamasina sp. 1, *M. mammifer*, and *C. binuseta* showed significant associations (*p* < 0.05) (Fig. 3). Among the taxa with significant associations, *Lasioseius* sp. showed an exponential increase in mean abundance with increasing beetle body area, suggesting a preference for larger hosts (Fig. 3A). In contrast, *M. mammifer* (Fig. 3B) showed a negative relationship, with smaller individuals harboring more mites. For Gamasina sp. 1 (Fig. 3C) and *C. binuseta* (Fig. 3D), abundance increased moderately with body size.

**Table 1:** Relationship between host body area (proxy: length × width) and abundance of different taxa of phoretic mites associated with *Rhynchophorus palmarum*

Taxon	Coefficient (β)	Standard error	t-statistic	*p*
*Lasioseius* sp.	0.00697	0.00214	3.26	0.001*
*Macrocheles mammifer*	-0.00957	0.00446	-2.14	0.033*
Gamasina sp. 1	0.00300	0.00145	2.08	0.039*
*Crenamargo binuseta*	0.00311	0.00157	1.97	0.049*
Uropodina sp. 1	-0.00332	0.00187	-1.77	0.077
*Fuscuropoda marginata*	0.00461	0.00278	1.66	0.098
Uropodidae sp. 1	-0.00174	0.00166	-1.05	0.295
Trematuridae sp. 1	-0.00226	0.00216	-1.05	0.296
*Centrouropoda* cf. *Brasiliana*	0.00110	0.00124	0.885	0.377
*Macrocheles* sp.	0.00169	0.00197	0.857	0.392
*Rhynchopolipus rhynchophori*	0.00115	0.00212	0.543	0.588
*Calvoliella* sp.	-0.00168	0.00851	-0.198	0.843
*Curculanoetus* sp.	0.000533	0.00321	0.166	0.868
*Dinychus* sp.	-0.0000935	0.00195	-0.0479	0.962

Values were estimated using generalized linear models (GLMs) with a quasi-Poisson distribution. Coefficients (β) represent the change in expected abundance associated with a unit increase in body area (mm²) and are expressed on the log scale. Significance: *p* < 0.05 (*)

### Temporal variation in the abundance of dominant phoretic mites

**Fig. 4:**
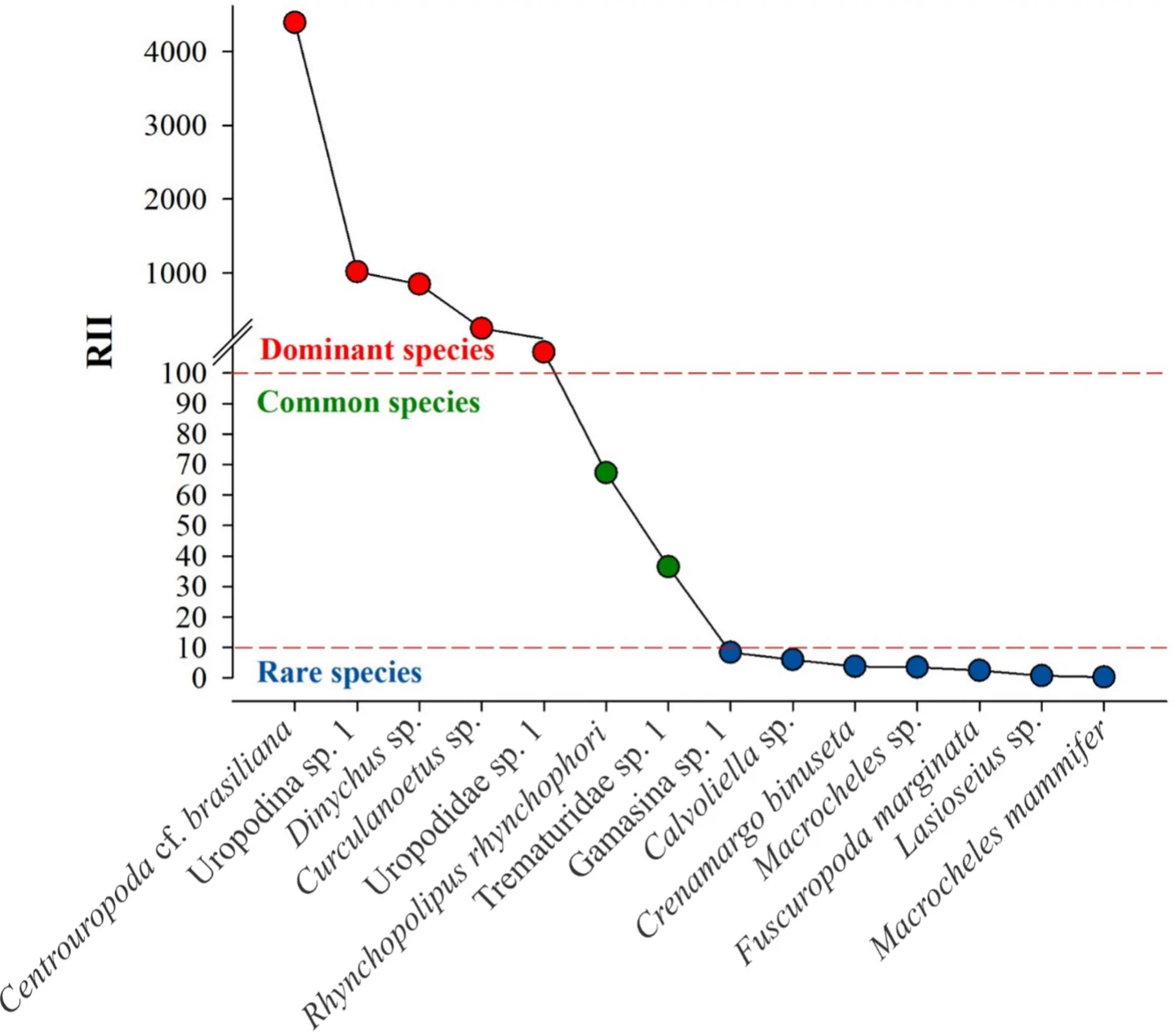
Distribution of mite taxonomic categories associated with *Rhynchophorus palmarum* based on the species importance index (SII). Species were classified as dominant (SII ≥ 100, in red), common (10 < SII < 100, in green), and rare (SII < 10, in blue). Dashed lines indicate the thresholds used for classification

**Fig. 5:**
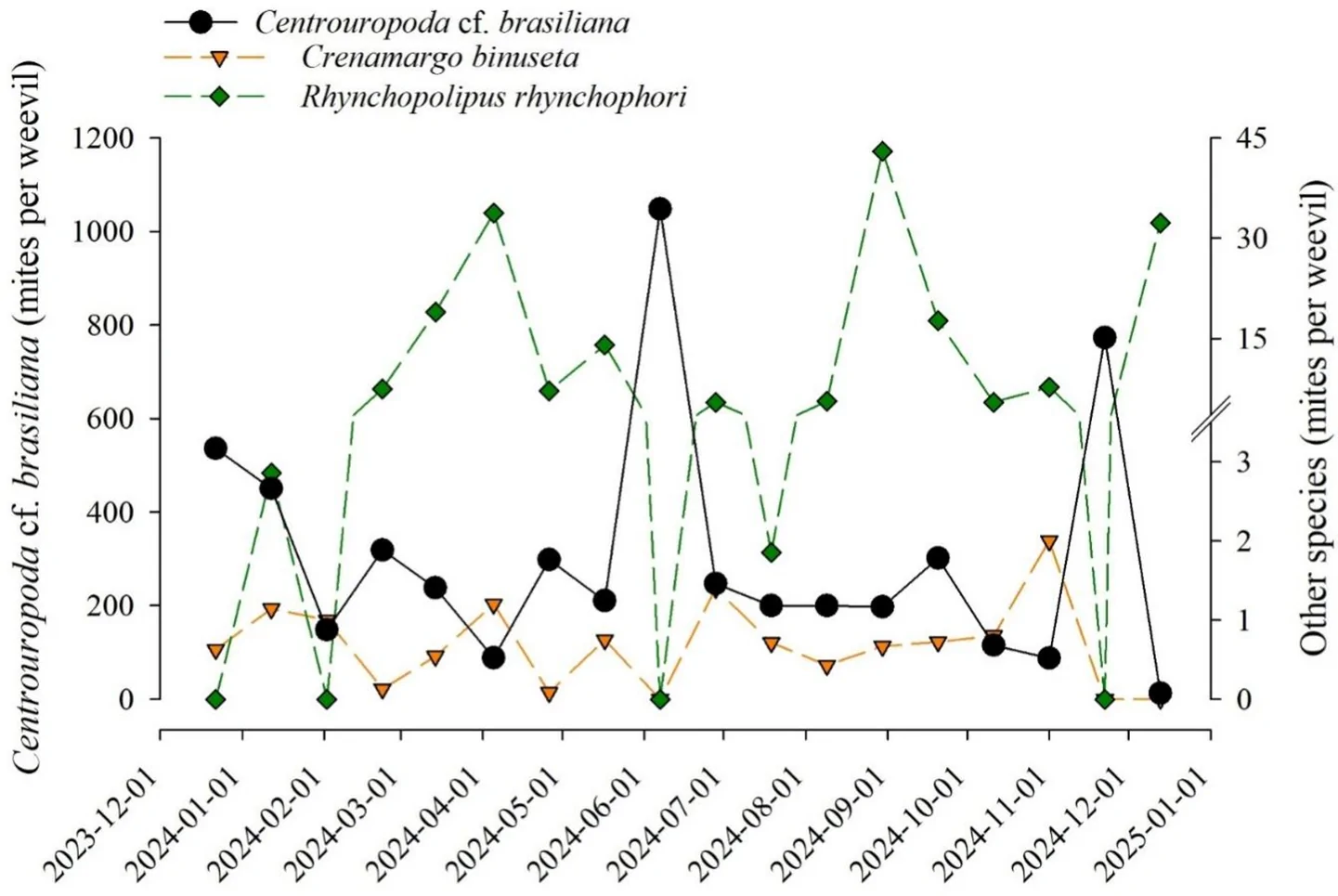
Temporal variation in the abundance of phoretic mites associated with *Rhynchophorus palmarum* in Igarassu, Pernambuco, Brazil. The abundance of *Centrouropoda* cf. *brasiliana* (left y-axis), the most dominant species, is shown in relation to the abundances of *Crenamargo binuseta* and *Rhynchopolipus rhynchophori* (right y-axis), which were included as predictor variables in the model explaining temporal changes in the abundance of *C.* cf. *brasiliana.* Data represent the mean number of mite specimens per beetle, sampled every 17 days over a one-year period

Among the 14 mite taxa recorded on *R. palmarum*, only five were classified as dominant (SII ≥ 100): *C.* cf. *brasiliana*, Uropodina sp. 1, *Dinychus* sp., *Curculanoetus* sp., and Uropodidae sp. 1 (Fig. 4). Because *C.* cf. *brasiliana* was the most dominant species, its temporal variation were analyzed. Approximately 42% of the population variation of *C.* cf. *brasiliana* can be explained by its (direct or indirect) interaction with *C. binuseta* (partial R² = 19.4) and *R. rhynchophori* (partial R² = 22.6) (F₂,₁₅ = 5.46; R² = 0.423; *P* = 0.016). Both predictors had negative effects on the mean abundance of *C.* cf. *brasiliana* (standardized β = −0.41 and − 0.47, respectively) (Fig. 5).

The mean number of *C.* cf. *brasiliana* specimens showed considerable variation throughout the year, with mean values per beetle ranging from 12.4 to 1,048 individuals. In general, periods of higher abundance of *C.* cf. *brasiliana* (e.g., June and December 2024) coincided with low densities of *R. rhynchophori*. Conversely, in sampling events where *R. rhynchophori* showed high values (e.g., 33.8; 43.0), the abundance of *C.* cf. *brasiliana* was considerably reduced (88.2 and 197.2, respectively). A similar pattern, although less pronounced, was observed for *C. binuseta*, whose presence at higher densities was also associated with reduced abundance of *C.* cf. *brasiliana*.

## Discussion

This study provides a comprehensive overview of the phoretic interaction between *R. palmarum* and its mite community, revealing consistent patterns of dominance, aggregation, and association with host traits. In the present study, it was observed that: (1) the 14 mite taxa associated with *R. palmarum* exhibited wide variation in prevalence, intensity, and abundance, but with a high aggregation index; (2) *C.* cf. *brasiliana* was the most prevalent and abundant species, reaching loads exceeding 2,800 individuals per host; (3) host sex and body size did not influence total phoretic load; however, body size may selectively influence the occurrence of specific taxa; and (4) the population fluctuation of *C.* cf. *brasiliana* was negatively influenced by the abundances of *C. binuseta* and *R. rhynchophori*, suggesting potential interspecific interactions modulating its temporal variation over time.

Our results reveal the high richness of phoretic taxa associated with *R. palmarum* in Brazil and provide novel evidence on variation in prevalence, intensity, and abundance, as well as demonstrating an aggregation pattern for these taxa. To date, the only systematic survey of phoretic mites associated with *R. palmarum* was conducted by Gómez-Marco et al. (2021), who documented three taxa (*Centrouropoda* sp. nov., *Dinychus* sp. nov., and *F. marginata*) in recently introduced populations in southern California. Prior to this, the only available records included the presence of two species (*C. almerodai* Hiramatsu & Hirschmann, 1992 and *Glyptholaspis* sp. Filipponi & Pegazzano (1960) (possibly *Centrouropoda* sp. nov. and *M. mammifer*, according to Gómez-Marco et al. (2021) and Dilipkumar et al. (2015), respectively) in occasional collections in Panama (Rodríguez-Morell et al. 2012), and records of *R. rhynchophori* and *C. binuseta* in Brazil, reported in taxonomic descriptions or isolated observations (Husband and Flechtmann 1972; Flechtmann 1981; Negrisoli Junior et al. 2011; Di Palma and Porcelli 2014). The higher richness observed in this study may reflect the long history of co-occurrence between *R. palmarum* and its symbiotic fauna in the Neotropics, the native range of the beetle. Furthermore, recently colonized areas may have undergone biotic filtering processes, resulting in partial loss of the associated mite fauna, as predicted by the Enemy Release Hypothesis (Williamson 1996).

All recorded taxa exhibited strongly aggregated distributions on hosts, with high values of Poulin’s discrepancy index. Similar patterns were reported by Matos et al. (2023) in phoretic mites associated with *R. ferrugineus* (Olivier, 1970), and may result either from uneven occupation of microhabitats (i.e., specific regions of the host body where mites attach, such as beneath the elytra or on the legs) or from competitive exclusion mechanisms driven by dominant species, such as *C.* cf. *brasiliana*. Alternatively, aggregation may reflect latent parasitic strategies, given that this pattern is also characteristic of parasites (Goater et al. 2014), and there are records of transitions from commensalism to active host exploitation among phoretic mites (Houck and Cohen 1995; Seeman and Walter 2023).

The mite *C.* cf. *brasiliana* stood out as the most prevalent and abundant taxon in the phoretic community associated with *R. palmarum*, being recorded on all sampled beetles and accounting for more than 70% of the individuals counted. Individual loads reached extreme values, exceeding 2,800 mites per host, which represents, to our knowledge, the highest record documented for phoretic mites on *R. palmarum*. Similar dominance patterns were observed by Gómez-Marco et al. (2021), in which *Centrouropoda* sp. nov. also stood out in abundance, especially in the subelytral region. More broadly, species of this genus have shown recurrent affinity with beetles of the genus *Rhynchophorus* in different regions of the world, such as *C. almerodai* on *R. ferrugineus* in Mediterranean and Southeast Asian countries (Dilipkumar et al. 2015). This broad distribution, combined with the high prevalence and abundance observed in different geographic contexts, suggests a strong ecological association between *R. palmarum* and mites of the genus *Centrouropoda*. However, the potential effects of this association on host biology remain poorly understood, and further investigation is needed to determine whether high loads, such as those documented in this study, result in physiological costs, as suggested for other *Rhynchophorus* species infested by Uropodina (Mazza et al. 2011).

In this study, neither sex nor body size of *R. palmarum* significantly influenced total phoretic load, suggesting that, these factors do not modulate the intensity of the association between beetles and mites. However, taxon-specific analyses revealed that body size may selectively affect the abundance of certain taxa, such as *Lasioseius* sp., Gamasina sp. 1, *C. binuseta*, and *M. mammifer*. The prevalence of these groups ranged from 6.5% to 39.7%, and the observed effects were of low magnitude, indicating that such associations should be interpreted with caution. These results partially align with those reported by Matos et al. (2023), who also found no differences in prevalence between males and females for seven taxa of phoretic mites associated with *R. ferrugineus*. In contrast, Dilipkumar et al. (2015) reported preferential association with males in *R. ferrugineus*, attributed to their larger body size and possible emission of chemical signals that may facilitate host selection by mites. Although the aggregation pheromone released by male *R. palmarum* is well known (Oehlschlager et al. 1995), there is no evidence that it functions as a kairomone for phoretic mites. Interestingly, Gómez-Marco et al. (2021) observed that infested males exhibited a slight reduction in pronotum width compared to non-infested individuals (≈ 3%), and suggested that this reduction may originate from earlier developmental stages, in the case where mites are associated with the larval or pupal stages of the beetle. Although the total phoretic load was not influenced by the body size of *R. palmarum*, some mite taxa responded significantly to this variable, indicating that host size may affect individuals taxa differently. Similar findings have been reported for highly specific phoretic systems involving burying beetles and uropodine mites. For example Bajerlein et al. (2026) found that host body size did not influence the prevalence or intensity of *Uroobovella nova* (Oudemans, 1902) (Urodinychidae) on *Nicrophorus beetles*, although infestation patterns differed among host species and between sexes. Together, these findings suggest that host body size alone is a poor predictor of phoretic mite load in highly specific associations, where host biology and species-specific ecological traits are likely more important determinants of infestation patterns.

The population fluctuation of *C.* cf. *brasiliana* over time showed peaks of abundance followed by sharp declines. Statistical models indicated that its abundance was negatively influenced by those of *C. binuseta* and *R. rhynchophori*, suggesting that interspecific interactions may modulate its dynamics. Although there are no previous studies directly testing this type of relationship among phoretic mites associated with *Rhynchophorus*, the aggregation and dominance patterns reported by (Matos et al. 2023; Gómez-Marco et al. 2021) point to possible mechanisms of competitive exclusion or microhabitat partitioning. In our observations, these three species occupy distinct regions on the host body: *C.* cf. *brasiliana* occurs in all regions, with predominance in the subelytral space; *R. rhynchophori* is restricted to the subelytral region; and *C. binuseta* occurs in all regions except the subelytral space. This spatial segregation suggests that the interactions observed in population fluctuations may be associated with spatial exclusion via microhabitat partitioning in the case of *C. binuseta*, and with direct overlap with *C.* cf. *brasiliana* in the case of *R. rhynchophori*, which could intensify competition for space or resources associated with the subelytral region. Additionally, although mites of the family Diplogyniidae are generally considered detritivores or microbivores, there is evidence that *C. binuseta* may exhibit facultative predatory behavior. Halliday (2019) proposed that this species may feed on phytoparasitic nematodes, such as *B. cocophilus*, or even impact the reproduction of *R. palmarum* by consuming eggs or larvae, although such interactions lack experimental validation. This reinforces the possibility that *C. binuseta* exerts direct or indirect effects on *C.* cf. *brasiliana*, either through competition or facultative exploitation of host-associated resources. Differences in presumed feeding habits among taxa may also contribute to their ecological segregation. Whereas *C.* cf. *brasiliana* is likely detritivorous or fungivorous (Uropodina), *C. binuseta* may exhibit detritivorous or opportunistic predatory habits (Diplogyniidae), and *R. rhynchophori* belongs to a family (Podapolipidae) that includes species with ectoparasitic habits, reinforcing the possibility of asymmetric or antagonistic interactions. Although abiotic factors and variation in host density cannot be ruled out, the results provide initial evidence that the composition of the phoretic community may influence the population trajectory of dominant taxa, revealing an additional level of complexity in mite–host interactions.

The results of this study reveal a structurally complex community of phoretic mites associated with *R. palmarum*, characterized by high diversity, strong aggregation, and asymmetric dominance patterns. The lack of significant influence of host sex and body size on total load suggests that mite colonization is more closely related to the biology of phoretic species than to morphological attributes of the beetle. However, specific associations between certain taxa and host size, as well as the negative influence of co-occurring species on the fluctuation of *C.* cf. *brasiliana*, indicate that subtle and spatially structured biotic interactions contribute to shaping community composition and dynamics. These findings highlight the importance of considering both host-related factors and interspecific interactions when investigating symbiotic associations, and pave the way for experimental studies to assess the functional and evolutionary consequences of these relationships.

## Supplementary Information

Below is the link to the electronic supplementary material.

Supplementary Material 1

## Data Availability

The datasets generated and/or analyzed during the current study are available from the corresponding author upon reasonable request.
