# Supplementary material for: Beyond phoresy: ecological patterns, effects of host attributes, and temporal variation in mites associated with Rhynchophorus palmarum L. (Coleoptera: Curculionidae)
Source: Exp Appl Acarol. 2026 Aug 24;97(3):29. doi: 10.1007/s10493-026-01171-6 (PMC13503439; doi:10.1007/s10493-026-01171-6)
Supplement: Supplementary file 1 [file 10493_2026_1171_MOESM1_ESM.docx]

**Beyond Phoresy: Ecological Patterns, Effects of Host Attributes, and Temporal Variation in Mites Associated with *Rhynchophorus palmarum* L. (Coleoptera: Curculionidae)**

Maria Beatriz Nunes de Souza¹, Maria Eduarda Barbosa Almeida², Erika Luiza dos Santos Torres², Hans Klompen³, Manoel Guedes Correa Gondim Junior^4^, Debora Barbosa de Lima¹, José Wagner da Silva Melo¹

¹Laboratório de Acarologia, Departamento de Zoologia, Programa de Pós-Graduação em Biologia Animal, Universidade Federal de Pernambuco, Recife, Pernambuco, Brazil

²Laboratório de Acarologia, Departamento de Zoologia, Graduação em Ciências Biológicas com ênfase em Ciências Ambientais, Universidade Federal de Pernambuco, Recife, Pernambuco, Brazil

³Department of Evolution, Ecology, and Organismal Biology, The Ohio State University, Columbus OH 43212, USA

^4^Laboratório de Acarologia, Departamento de Agronomia, Programa de Pós-Graduação em Entomologia, Universidade Federal Rural de Pernambuco, Recife, Pernambuco, Brazil

Corresponding author: m-beatriz2007@hotmail.com

Journal: Experimental and Applied Acarology

**Supplementary Table 1** Prevalence, mean infestation intensity (MI) and mean abundance (MA) of phoretic mites associated with males (M) and females (F) of *Rhynchophorus* *palmarum*

| Taxa | Sex | P (n) ^a^ | Prev^b^ (CI 95%)^c^ | MI^d^ (CI 95%)^e^ | MA^f^ (CI 95%)^e^ |
| --- | --- | --- | --- | --- | --- |
| *Calvoliella* sp. | M | 8 (112) | 0,071 (0.031-0.136) | 148 (23-617.81) | 10.57 (1.27-53.81) |
|  | F | 1(87) | 0.011 (0-0.062) | 191 (-) | 2.2 (0-6.59) |
| *Centrouropoda* cf. *brasiliana* | M | 101 (112) | 0.902 (0.831-0.95) | 257.2 (199.12-338.4) | 231.94 (180.37-305.06) |
|  | F | 74 (87) | 0.851 (0.758-0.918) | 344.49 (251.55-479.77) | 293.01 (206.08-416.72) |
| *Crenamargo binuseta* | M | 41(112) | 0.366 (0.277-0.462) | 2.05 (1.63-2.52) | 0.75 (0.54-1.04) |
|  | F | 20 (87) | 0.23 (0.146-0.332) | 2.1 (1.58-2.99) | 0.48 (0.29-0.77) |
| *Curculanoetus* sp. | M | 28 (112) | 0.25 (0.173-0.341) | 218.54 (119.8-404.9) | 54.63 (28.5-106.46) |
|  | F | 25 (87) | 0.287 (0.195-0.394) | 134.88 (61.83-305.6) | 38.76 (15.35-95.75) |
| *Dinychus* sp. | M | 55 (112) | 0.491 (0.395-0.587) | 158.02 (102.14-259.67) | 77.6 (47.91-133.44) |
|  | F | 47 (87) | 0.54 (0.43-0.648) | 175.38 (119.84-248.03) | 94.75 (64.31-142.8) |
| *Fuscuropoda marginata* | M | 22 (112) | 0.196 (0.127-0.282) | 3.59 (2.05-5.95) | 0.71 (0.36-1.35) |
|  | F | 12 (87) | 0.138 (0.073-0.229) | 5.92 (2.84-11.83) | 0.82 (0.3-2.02) |
| Gamasina sp. 1 | M | 38 (112) | 0.339 (0.253-0.435) | 2.26 (1.79-3.06) | 0.77 (0.53-1.11) |
|  | F | 41 (87) | 0.471 (0.363-0.581) | 3.2 (2.49-4.05) | 1.51 (1.05-2.07) |
| *Lasioseius* sp. | M | 17 (112) | 0.152 (0.091-0.232) | 1.59 (1.18-2.29) | 0.24 (0.14-0.43) |
|  | F | 11 (87) | 0.126 (0.065-0.215) | 2.18 (1.36-3.36) | 0.28 (0.13-0.54) |
| *Macrocheles mammifer* | M | 10 (112) | 0.089 (0.044-0.158) | 2.0 (1.4-2.7) | 0.18 (0.08-0.33) |

|  | F | 3 (87) | 0.034 (0.007-0.097) | 6.33 (1-11.67) | 0.22 (0.01-1.15) |
| --- | --- | --- | --- | --- | --- |
| *Macrocheles* sp. | M | 29 (112) | 0.259 (0.181-0.35) | 3.03 (2.31-4.34) | 0.79 (0.48-1.21) |
|  | F | 19 (87) | 0.218 (0.137-0.32) | 3.21 (2.21-4.47) | 0.70 (0.39-1.18) |
| *Rhynchopolipus rhynchophori* | M | 30 (112) | 0.268 (0.189-0.36) | 37.1 (26.9-54.09) | 9.94 (6.12-16.18) |
|  | F | 30 (87) | 0.345 (0.246-0.454) | 39.33 (25.03-65.26) | 13.56 (7.7-23.38) |
| Trematuridae sp. 1 | M | 46 (112) | 0.411 (0.319-0.508) | 10.13 (7.24-18.07) | 4.16 (2.77-7.63) |
|  | F | 37 (87) | 0.425 (0.32-0.536) | 11.73 (7.22-19.94) | 4.99 (2.99-9.16) |
| Uropodidae sp. 1 | M | 77 (112) | 0.688 (0.593-0.772) | 11.74 (8.74-20.17) | 8.07 (5.94-14.18) |
|  | F | 65 (87) | 0.747 (0.643-0.834) | 9.8 (7.46-13.12) | 7.32 (5.36-9.91) |
| Uropodina sp. 1 | M | 65 (112) | 0.58 (0.483-0.673) | 123.86 (92.86-170.58) | 71.88 (50.82-104.58) |
|  | F | 49 (87) | 0.563 (0.453-0.669) | 207.08 (127.61-338.31) | 116.63 (70.07-199.95) |

^a^Number of infested beetles followed by the total number of examined beetles in parentheses

^b^Prevalence (proportion of infested beetles) with 95% confidence interval (CI)

^c^CIs calculated based on the exact binomial distribution

^d^Mean infestation intensity (average number of mites among infested beetles), with 95% CI

^e^Confidence intervals for MI and MA obtained via bootstrap (n = 2,000 replicates)

^f^Mean abundance (average number of mites considering all examined hosts), with 95% CI
